# Teachers' Attitude Toward Situations of Dental Trauma in a Capital in Southeast Brazil

**DOI:** 10.1111/edt.13076

**Published:** 2025-06-09

**Authors:** Pamela Barbosa dos Santos, Vanessa Felipe Vargas‐Moreno, Gilda Rocha dos Reis‐Neta, Altair Antoninha Del Bel Cury, Maria Helena Monteiro de Barros Miotto

**Affiliations:** ^1^ Department of Prosthodontics and Periodontology, University of Campinas (UNICAMP) Piracicaba Dental School Piracicaba SP Brazil; ^2^ Department of Clinical Dentistry Federal University of Espírito Santo (UFES) Vitória Espírito Santo Brazil

**Keywords:** dental trauma, prevalence, teachers

## Abstract

**Introduction:**

Teachers play a crucial role in first aid for dental trauma (DT), as children spend a large part of their time in schools. In these environments, accidents are frequent. Immediate and correct intervention is essential for prognosis.

**Aim:**

To assess the attitudes of teachers in the municipal school system in the municipality of Vitória‐ES towards different DT scenarios and to associate them with socioeconomic, demographic, and functional variables.

**Material and Methods:**

A quantitative, cross‐sectional, and probabilistic study was carried out. Data was collected using self‐administered and validated questionnaires, covering sociodemographic and functional information and teachers' knowledge of DT. To this end, teachers' attitudes were classified as correct or incorrect and assessed considering three different DT scenarios: 1—crown fracture; 2—lateral luxation; 3—tooth avulsion. Descriptive analyses, frequency tables, and statistical analyses using the chi‐square test (*p* < 0.05) were carried out. A total of 292 teachers from 37 schools took part in the study.

**Results:**

Among the participants, 137 (46.9%) teachers had already witnessed cases of DT, 94 (68.6%) of which had occurred in the school environment. In addition, 274 (93.8%) of the teachers had never received formal guidance. In scenario 3 (tooth avulsion), only 18.3% of teachers answered correctly. Teachers who had received training in dental trauma had (33.3%) correct answers and (66.7%) incorrect answers. Teachers who had not received training got (13.1%) correct, while (86.9%) got it wrong. This difference was statistically significant (*p* = 0.03). Similarly, teachers who had experienced dental trauma showed a potentially significant association (*p* = 0.05) with correct attitudes.

**Conclusion:**

There was a high frequency of inappropriate behavior in the event of tooth avulsion, highlighting the need for first aid training. This reinforces the need for educational interventions in the school environment, aimed at providing adequate training in the correct handling of dental emergencies.

## 1 Introduction

Dental trauma (DT) is defined as unpredictable injuries caused by external impact on one or more teeth, the periodontium, and surrounding soft tissues [1]. These events occur frequently in children and adolescents, accounting for approximately 5% of all injuries [2]. Among adults, 33% reported trauma to their permanent teeth, with the majority recorded before the age of 19 [2]. The high prevalence of these injuries highlights their relevance as a public health problem [1, 3, 4, 5]. Tooth loss resulting from trauma can have lifelong consequences [6, 7, 8], including aesthetic, functional, and psychological repercussions that negatively impact the individual's quality of life [4, 9]. Given the vulnerability of specific age groups, special attention should be paid to young children, who are at greater risk of dental trauma.

The preschool age group is particularly prone to TD [10]. Therefore, parents, teachers, and caregivers of children should be aware of the immediate measures to be taken to improve prognosis and expand treatment options [10, 11]. Considering that children spend a significant part of their time at school, dental injuries in this environment are relatively common [12]. First aid actions play a crucial role, especially in cases of tooth avulsion [13]. Immediate replantation or proper transportation of the avulsed tooth can significantly improve the prognosis [11]. Teachers are often the first to witness DT situations [14, 15]. Mastering basic knowledge on the subject is essential for them to understand the consequences of inadequate actions in emergency management, increasing their safety, motivation, and preparedness to care for a child victim of TD [16, 17].

Studies in different parts of the world indicate that elementary school teachers often lack adequate knowledge about the proper management of dental trauma emergencies [10, 12]. To date, no study has been carried out in the city of Vitória–ES, to assess the attitudes of elementary school teachers in municipal public schools towards cases of dental trauma. Considering the impact of dental trauma (DT) on children's health and the crucial role that teachers can play in the initial management of these situations, this study assessed the attitudes of teachers in the municipal school system of Vitória–ES towards different scenarios of crown fracture, lateral luxation, and dental avulsion, and associated them with socioeconomic, demographic, and functional variables.

## 2 Materials and Methods

This study was approved by the Research Ethics Committee (65870322.0.0000.5060). Participation was voluntary and the answers to the questionnaire were processed anonymously, following all the ethical principles of the Declaration of Helsinki.

This is a cross‐sectional observational study, with a quantitative design, carried out between February and December 2023, in the municipality of Vitória, Espírito Santo, with teachers from the municipal elementary school system. The inclusion criteria were being a teacher working in the municipal elementary school system in Vitória–ES. The exclusion criteria were teachers on sick leave or maternity leave. A sample was calculated with a prevalence of 50%, an error of 5%, and a 95% confidence interval, resulting in an initial sample of 315 teachers. Considering a possible loss of up to 8% of participants, a final sample of 292 randomly selected teachers was obtained.

To carry out the randomization, a list provided by the Human Resources department of the Vitória City Hall was used. This list gave the total number of teachers in each school in the municipal network. Based on the sample of 315 teachers, the number of participants per school unit was calculated proportionally. This process ensured that the sample was diversified and randomized. Some permanent teachers worked in different schools, varying between the morning and afternoon shifts.

Data was collected using validated and adapted questionnaires, which were applied in person using a self‐administered technique [18]. The first questionnaire assessed the socioeconomic status of the participants based on the Brazil Economic Classification Criterion, developed by the Brazilian Association of Research Companies (ABEP) [19]. The second questionnaire, called “Functional”, contained 15 questions covering age, place of work, level of education, length of professional experience, and closed questions on knowledge and expertise related to dental trauma. Finally, the third questionnaire was dedicated to specific aspects of DT, which featured images accompanied by objective questions, including three scenarios of DT in the permanent dentition, in which the teachers had to select the most appropriate attitude considering the clinical cases presented.

To assess teachers' knowledge in the third questionnaire, the criteria for classifying answers as correct or incorrect were based on the International Association of Dental Trauma guidelines, version 2020 [2, 6]. The answers were categorized into correct or incorrect attitudes, the latter being considered capable of compromising the prognosis in three scenarios: scenario 1 presented the clinical case of a 9‐year‐old boy who fell, resulting in a fracture of the enamel and dentin at the apex of the central incisor; scenario 2 referred to a 12‐year‐old student who suffered a lateral dislocation with two displaced upper teeth; scenario 3 dealt with a 12‐year‐old adolescent who fell down a flight of stairs, resulting in the avulsion of the upper central incisor.

Statistical analyses were done using the IBM SPSS software package, version 20. They included a descriptive analysis of the data presented in frequency tables with absolute and relative values, covering socioeconomic, functional, and dental trauma‐related variables. Chi‐square statistical tests were applied to assess associations between independent and dependent variables. The significance level adopted was (*p* < 0.05).

## 3 Results

The study population included 1727 active teachers from the municipal elementary school network, distributed across 52 schools. One school was excluded due to temporary closure for renovations. Of the remaining 51 schools, 12 refused to participate, often citing scheduling conflicts. All the schools were visited, and 37 (71.15%) agreed to take part after discussions with principals and pedagogues. The final sample consisted of 292 teachers, with no loss of participants. The sociodemographic and functional data of the sample are shown in Table 1, and the teachers' knowledge of dental trauma is shown in Table 2.

**TABLE 1:** Socio‐demographic and functional data of elementary school teachers.

	Number	Percentage (%)
Sex		
Male	69	23.6
Female	223	76.4
Age group		
up to 39 years old	102	34.9
40–49 years	89	30.5
≥ 50 years	101	34.6
Marital status		
Single	110	37.7
Married/stable union	131	44.8
Separated/widowed/other	51	17.5
Socio‐economic status		
A	33	11.3
B1‐B2	211	72.2
C1‐C2	48	16.5
Living with children		
Yes	159	54.5
No	43	14.7
No children	90	30.8
Time since graduation		
Up to 10 years	90	30.8
11–20 years	117	40.1
≥ 21 years old	85	29.1
Level of training		
Graduation	50	17.1
Specialization	212	72.6
Master's—Doctorate	30	10.3
Length of professional experience		
Up to 10 years	96	32.9
11–20 years	109	37.3
≥ 21 years old	87	29.8
Activities that accompany students		
At playtime	95	32.5
Out‐of‐class activities	129	44.2
Sports and others	68	23.3
Total	292	100

**TABLE 2:** Kindergarten teachers' perception of dental trauma.

	Number	Percentage (%)
Has received first aid training		
Yes	75	25.7
No	217	74.3
Self‐knowledge about dental trauma		
Good	14	4.8
Average	19	6.5
Regular	26	8.9
Bad	82	28.1
None	151	51.7
Received guidance on dental trauma		
Yes	18	6.2
No	274	93.8
Witnessed dental trauma		
Yes	137	46.9
No	155	53.1
Place where you witnessed the dental trauma		
Home	16	11.7
School	94	68.6
Street	12	8.8
Others	15	10.9
Measures taken if the student has suffered dental trauma		
Immediate, on‐the‐spot action	72	24.7
See your dentist	75	25.7
I would say to the parents	127	43.4
Everything that precedes	18	6.2
Would you feel safe providing first aid in the event of dental trauma?		
Yes	31	10.6
No	261	89.4
How do you rate your knowledge of dental trauma treatment?		
Very important	222	76.0
Important	61	20.9
Indifferent	6	2.1
It's not important	3	1.0
Unnecessary	0	0.0
Total	292	100

Table 3 shows the results for scenarios 1, 2, and 3.

**TABLE 3:** Relationship between teachers' correct and incorrect attitudes in scenarios 1, 2, and 3 and their relationship with demographic and functional variables, and teachers' knowledge of dental trauma.

	Scenario 1			Scenario 2			Scenario 3		
	The right attitude	Wrong attitude	*p*	The right attitude	Wrong attitude	*p*	The right attitude	Wrong attitude	*p*
	*N* (%)	*N* (%)		*N* (%)	*N* (%)		*N* (%)	*N* (%)	
Sociodemographic and economic variables									
Sex									
Male	38 (55.1)	31 (44.9)	0.343	50 (72.5)	19 (27.5)	0.379	12 (17.4)	57 (82.6)	0.263
Female	131 (58.7)	92 (41.3)		155 (69.5)	68 (30.5)		30 (13.5)	193 (86.5)	
Age group									
Up to 39 years old	62 (60.8)	40 (39.2)	0.270	76 (74.5)	26 (25.5)	0.148	13 (12.7)	89 (87.3)	0.345
≥ 40 years	107 (56.3)	83 (43.7)		129 (67.9)	61 (32.1)		29 (15.3)	161 (84.7)	
Socio‐economic status									
AB	143 (58.6)	101 (41.4)	0.339	173 (70.9)	71 (29.1)	0.335	36 (14.8)	208 (85.2)	0.442
CD	26 (54.2)	22 (45.8)		32 (66.7)	16 (33.3)		6 (12.5)	42 (87.5)	
Time since graduation									
Up to 10 years	48 (53.3)	42 (46.7)	0.178	63 (70.0)	27 (30.0)	0.532	14 (15.6)	76 (84.4)	0.414
> 10 years	121 (59.9)	81 (40.1)		142 (70.3)	60 (29.7)		28 (13.9)	174 (86.1)	
Length of professional experience									
Up to 10 years	53 (55.2)	43 (44.8)	0.301	67 (69.8)	29 (30.2)	0.509	14 (14.6)	82 (85.4)	0.538
> 10 years	116 (59.2)	80 (40.8)		138 (70.4)	58 (29.6)		28 (14.3)	168 (85.7)	
Functional variables									
First aid training									
Yes	45 (60.0)	30 (40.0)	0.385	56 (74.7)	19 (25.3)	0.203	12 (16.0)	63 (84.0)	0.385
No	124 (57.1)	93 (42.9)		149 (68.7)	68 (31.3)		30 (13.8)	187 (86.2)	
Received first aid advice in the event of dental trauma									
Yes	11 (61.1)	7 (38.9)	0.489	16 (88.9)	2 (11.1)	0.056	6 (33.3)	12 (66.7)	0.030*
No	158 (57.7)	116 (42.3)		189 (69.0)	85 (31.0)		36 (13.1)	238 (86.9)	
Witnessed dental trauma									
Yes	84 (61.3)	53 (38.7)	0.159	98 (71.5)	39 (28.5)	0.368	20 (14.6)	117 (85.4)	0.526
No	85 (54.8)	70 (45.2)		107 (69.0)	48 (31.0)		22 (14.2)	133 (85.8)	
Knowledge of dental trauma									
Good‐Bad	21 (63.6)	12 (36.4)	0.302	26 (78.8)	7 (21.2)	0.174	6 (18.2)	27 (81.8)	0.331
None	148 (57.1)	111 (42.9)		179 (69.1)	80 (30.9)		36 (13.9)	223 (86.1)	
Would you feel safe providing first aid in the event of dental trauma?									
Yes	21 (67.7)	10 (32.3)		24 (77.4)	7 (22.6)	0.239	7 (22.6)	24 (77.4)	0.136
No	148 (56.7)	113 (43.3)	0.163	181 (69.3)	80 (30.7)		35 (13.4)	226 (86.6)	
(%)	57.4	42.6		70.4	29.6		18.3	81.7	

* Chi‐square test (*p*‐value < 0.05).

It shows the associations between sociodemographic and socioeconomic variables (gender, age group, socioeconomic status, length of training and professional experience).

It also relates these variables to functional variables (guidance on first aid, instructions on dental trauma, self‐perceived knowledge and self‐confidence). Compared to the other scenarios, scenario 2 shows the highest proportion of correct attitudes at 70.4%. However, most teachers had not received training in first aid or guidance on trauma when evaluating this scenario. In scenario 3, only 18.3% of teachers had correct attitudes, while 81.7% gave incorrect answers.

## 4 Discussion

Mastering the management of DT is crucial to providing adequate care for injured children and optimizing the prognosis of affected teeth. The prognosis in cases of DT is closely related to the time and previous attitudes adopted at the scene of the accident [2, 6]. Therefore, this study assessed the attitude of teachers from the municipal school system in Vitória—ES when faced with three scenarios of crown fracture, lateral luxation, and tooth avulsion. In scenario 3, it was observed that teachers who had received some prior knowledge on the subject had a higher rate of correct answers when compared to those who had not received any guidance on the subject. However, they still had high rates of incorrect attitudes. This finding is worrying, considering that tooth avulsion is one of the most serious injuries to permanent teeth. Furthermore, the prognosis for avulsed permanent teeth is directly influenced by the immediate actions taken at the scene of the accident [14].

The lack of adequate training on DT among teachers further highlights the need for structured educational interventions to improve decision‐making at the accident scene. In the functional questionnaire, 18 (6.2%) teachers received guidance on dental trauma, a result consistent with studies that reported that only (4%), (4.1%), (7.6%), (10%), (15%), and (26%) of teachers received guidance on the subject, respectively [12, 13, 18, 20, 21, 22]. As evidenced by a study carried out in India, educational programs have the potential to promote a significant improvement in knowledge about TD among teachers after the implementation of training courses [23]. When considering previous experience with TD, it was found that (46.9%) of the teachers had already witnessed occurrences of TD, especially at school (68.6%). These results corroborate previous studies in which (16.6%), (50.7%) and (63%) of the participants had already witnessed a situation involving TD [16, 20, 24]. Demonstrating how common the problem is among preschoolers and highlighting how this experience can significantly impact the management of the situation. Furthermore, since children spend a considerable amount of time at school daily, dental injuries are more likely to occur in this environment [18, 22]. This reality highlights the extreme importance of the role of teachers.

When asked how they would deal with a case of TD, only 25.7% of the teachers said they would refer the student to the dentist. This rate is similar to that observed in other studies, in which (37.4%) and (50%) of teachers opted to seek medical/dental care immediately [20, 25]. Furthermore, in a 2023 survey, the majority of teachers (86.1%) considered that the educator's immediate attitude is essential to save the tooth [16]. It was also noted that (43.4%) of teachers would expect parents to take the initiative. These responses together highlight the need to train teachers to seek immediate dental care, since time is an essential prognostic factor for successful treatment [18, 22]. The restriction on leaving school, imposed in cases of dental emergencies, represents a significant challenge. Until the parents are contacted and arrive at the school, a considerable amount of time is lost. This delay can compromise the prognosis of dental trauma.

When asked about their self‐perceived level of knowledge, most teachers reported that it ranged from no knowledge to knowledge considered inadequate, totaling 79.8%. These results are consistent with studies in which a low perception of expertise in the management of DT was also observed [18, 20, 26]. The lack of preparation is not limited to the dental field, as (25.7%) of teachers said they had received first aid training to deal with health problems in general. These data are similar to the findings of a cross‐sectional survey, which revealed that only 22% and 29% of elementary school teachers had received formal training in first aid [12, 21]. A study carried out in the southern region of Brazil found that the lack of training in first aid contributes to the low level of knowledge in DM [27].

When asked about the relevance of DT‐related knowledge, most teachers (76.0%) considered it “Very Important”. This perception may be associated with the high prevalence of dental trauma cases witnessed by teachers. This recognition opens up opportunities for the development of training courses. These data corroborate the findings of several studies, which indicate that a significant proportion of teachers (86%), (84%), and (93.7%) expressed an interest in receiving training in the management of DT [20, 21, 24].

When comparing the findings of this study with research carried out in other Brazilian states [16, 20, 28], a low level of knowledge in the management of dental trauma was also observed. Given this, it is recommended that public health policies focused on teachers' health literacy be developed, implemented, and strengthened. These actions aim to improve the prognosis and well‐being of affected children in the school environment. In addition, schools offer a direct channel for reaching students and parents, allowing preventive measures to be discussed from an early age [29].

Although this study achieved the proposed objectives, the results refer to a limited population located in the capital of one state. More research is needed to assess and compare knowledge, practice, and attitudes towards emergency management in DT in other regions, providing a broader perspective. When asked about the topic's relevance, the majority of participants highlighted it as very important; however, it is possible that some felt inhibited by the presence of the researcher, promoting an information bias. Furthermore, more studies must be carried out to analyze teachers' levels of knowledge following the implementation of training courses to assess the effectiveness of learning.

The statistically significant association between the few teachers who received guidance on TD and their relationship with correct attitudes highlights the importance of training for a better prognosis. Lectures, courses, posters, apps, comics, realistic theaters simulating DT, information folders, and continuing teacher training are effective strategies for health education [20, 30, 31]. In this context, political leaders should consider implementing health literacy programs that promote greater awareness and preparation on the subject [29]. Therefore, TD management and first aid in teacher training curricula are necessary to provide adequate preparation for dealing with emergency situations [21, 23].

## 5 Conclusion

The study revealed that teachers in Vitória's municipal school system have a low rate of correct attitudes toward cases of tooth avulsion. In addition, the teachers who received information on dental trauma showed better behavior when faced with trauma, which suggests the need for structured and mandatory educational programs aimed at teachers.

## Author Contributions

The authors take full responsibility for this article.

## Conflicts of Interest

The authors declare no conflicts of interest.

## Data Availability

The data that support the findings of this study are available from the corresponding author upon reasonable request.
